# Mechanical thrombectomy for mild stroke with anterior circulation large vessel occlusion: a multicenter cohort study

**DOI:** 10.3389/fneur.2025.1668704

**Published:** 2025-09-05

**Authors:** Gaopan Zhang, Neng Zhang, Jingfan Li, Siyu Zhang, Manhe Li, Xiongfei Zhao

**Affiliations:** ^1^Department of Neurology, Xianyang Hospital of Yan'an University, Xianyang, China; ^2^Department of Neurosurgery, The First Hospital of Yulin and The Second Affiliated Hospital, Yan'an University, Yulin, China; ^3^School of Clinical Medicine, Xi'an Medical University, Xi'an, China

**Keywords:** ischemic stroke, thrombectomy, intracranial hemorrhages, mortality, odds ratio

## Abstract

**Background:**

The clinical benefit of mechanical thrombectomy (MT) for anterior circulation emergency large vessel occlusion (ELVO) in patients presenting with a mild National Institutes of Health Stroke Scale score (<6) remains uncertain. We aimed to assess the efficacy and safety of MT in this patient population.

**Methods:**

We enrolled individuals presenting with mild stroke attributable to anterior circulation LVO from three stroke centers between March 2020 and June 2024. The primary endpoint was an excellent 90-day outcome, defined as a modified Rankin Scale (mRS) score of 0–1. Functional independence at day 90 (mRS 0–2) was considered the secondary endpoint. Safety endpoints consisted of symptomatic intracranial hemorrhage (sICH) and all-cause mortality within 90 days. Multivariable logistic regression with inverse probability of treatment weighting (IPTW) was applied to examine the association between MT and clinical outcomes.

**Results:**

In total, 140 individuals with mELVO were selected for analysis, receiving either MT [*n* = 48; 35 males; mean age: 59.9 years; NIHSS median: 4 (IQR 2–5)] or medical management (MM) [*n* = 92; 62 males; mean age: 61.9 years; NIHSS median: 3 (IQR 1–4)]. No statistically significant differences were observed between the MT and MM groups in excellent outcome (aOR = 0.93; 95% CI, 0.41–2.11), functional independence (aOR = 2.14; 95% CI, 0.77–5.91), symptomatic intracranial hemorrhage (aOR = 1.63; 95% CI, 0.37–7.14), or mortality (aOR = 0.56; 95% CI, 0.02–20.94). The results remained consistent after IPTW adjustment.

**Conclusion:**

Among patients with mELVO, MT was not associated with significantly different outcomes compared to MM. Further investigation through randomized controlled trials is warranted.

## Introduction

Several randomized controlled studies have been demonstrated that mechanical thrombectomy (MT) leads to improved neurological outcomes in patients with anterior circulation emergency large vessel occlusion (ELVO), compared to medical management (MM) (1–4). As such, the American Heart Association (AHA) recommends that MT be considered the recommended treatment for anterior circulation ELVO and National Institutes of Health Stroke Scale (NIHSS) scores of at least 6 (5, 6). Patients with ELVO with a NIHSS < 6 (mELVO) were commonly excluded from these trials, with only three studies including a limited number of patients with mild neurological deficits (7–9).

As many as 10% of patients with ELVO demonstrate only minor stroke symptoms (10–12). Approximately 25% of patients with low-NIHSS ELVO stroke who received medical management did not achieve functional independence at 3 months (13). Only a small proportion of those receiving intravenous thrombolysis (IVT) achieved successful recanalization (14). A considerable number of patients with mild ELVO stroke may still experience poor clinical outcomes. Therefore, MT might offer greater functional benefit for individuals presenting with mild neurological symptoms.

Evidence supporting the clinical benefit and risks of MT in this specific subgroup remains limited. Therefore, we performed a multicenter retrospective study to assess the safety and efficacy outcomes of MT or MM in patients with mELVO.

## Methods

### Study participants

Between March 2020 and June 2024, individuals with mELVO who met the following criteria were identified from individuals admitted to three stroke centers:(1) age ≥ 18 years;(2) anterior circulation ELVO (internal carotid artery, M1 middle cerebral artery, and M2 middle cerebral artery) received treatment within 24 h after symptom emergence; (3) baseline NIHSS below 6. Patient data were retrieved via the hospital's electronic record system. For those undergoing MT, the preprocedural NIHSS score was applied; for MM patients, the admission score was used. Patients who had an NIHSS score of ≤ 5 at admission but experienced clinical deterioration resulting in a preprocedural NIHSS score >5 and subsequently underwent MT were removed from analysis. The MM group comprised patients who received either IVT alone or conservative treatment, whereas the MT group included those treated with MT alone or with bridging IVT. Patients with incomplete baseline data were excluded from the statistical analysis.

### Study treatments and interventions

We collected baseline data including age, sex, history of hypertension, diabetes, hyperlipidemia, atrial fibrillation, coronary artery disease, smoking status, Alberta Stroke Program Early Computed Tomography (CT) Score, modified Rankin Scale (mRS) and NIHSS scores at admission, site of vessel occlusion, IVT, and the time from last seen well to treatment.

### Outcomes measures

We evaluated the following efficacy outcomes: the primary end point was 90-day excellent outcome (defined as mRS score of 0–1). The secondary end point was 90-day functional independence (defined as mRS score of 0–2). Safety outcomes included 90-day mortality and symptomatic intracranial hemorrhage (sICH) (15). The mRS at 90 days was evaluated by study personnel through face-to-face or telephone follow-up.

### Statistical analysis

Normality of continuous variables was assessed using the Shapiro–Wilk test. Data that were normally distributed were summarized as mean (SD), while non-normally distributed data were reported as median and interquartile range (IQR). Categorical data were expressed as proportions (%). Group differences in continuous variables were analyzed using the Wilcoxon-Mann-Whitney test, and categorical data were examined using either the chi-square or Fisher exact test, depending on applicability. In addition, inverse probability of treatment weighting (IPTW) was applied to balance baseline differences between the two groups. To evaluate covariate balance after weighting, we calculated standardized mean differences (SMD), considering values below 0.20 as acceptable.

Additionally, multivariable logistic regression was performed with adjustment for sex, age, baseline NIHSS score, and occlusion site to estimate adjusted odds ratios (aORs) with 95% confidence intervals (CIs). The relationship between MT and clinical outcomes was assessed using logistic regression adjusted with IPTW.

### Subgroup analyses

To explore the potential heterogeneity in treatment efficacy across different treatment windows, patients were stratified into early (≤6 h) and late (6–24 h) subgroups. Multivariable logistic regression was used, adjusting for sex, age, baseline NIHSS score, and occlusion site.

IBM SPSS Statistics 27.0 (IBM Corp., Armonk, NY) was used for general statistical analysis, and RStudio software (version 4.4.3) was applied for building and evaluating the IPTW model. Statistical significance was determined using a two-sided threshold of *P* < 0.05.

## Results

### Baseline characteristic

**Figure 1:**
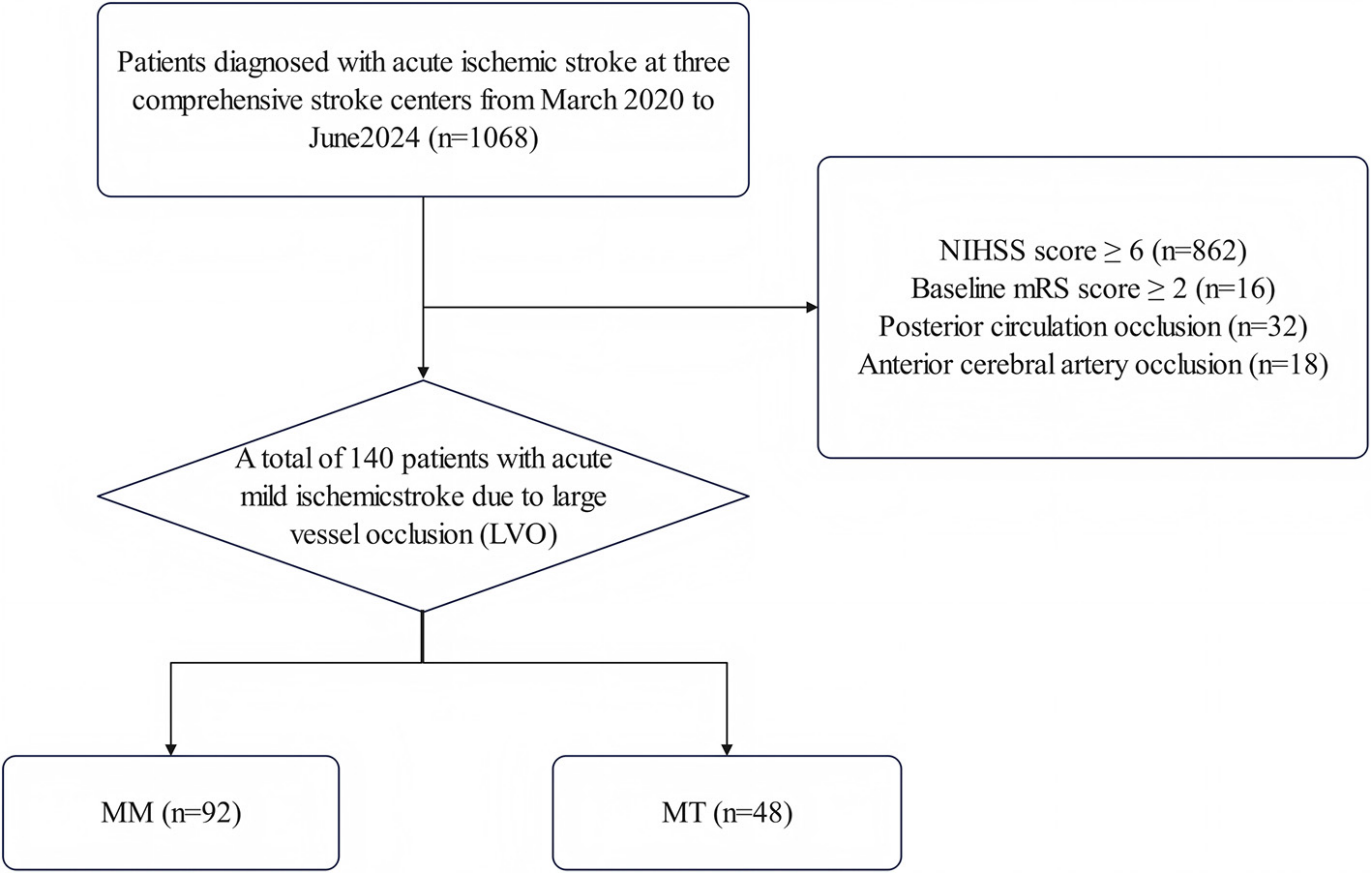
Flow of patients in the study.

A total of 140 patients with mELVO were included based on established selection criteria. The study flowchart is presented in Figure 1. 48 patients were treated with MT [mean (SD) age, 59.9 (12.2) years; 72.9% male; median NIHSS 4 (IQR 2–5)], and 92 received MM [mean (SD) age, 61.9 (10.4) years; 67.4% male; median NIHSS 3 (IQR 1–4)]. Baseline variables for the MT and MM groups are summarized in Table 1. Compared to MM, MT showed a greater proportion of internal carotid artery (ICA) occlusions (50% vs. 19.6%) and lower proportions of MCA-M1 (43.8% vs. 63%) and MCA-M2 (6.3% vs. 17.4%) occlusions (*P* < 0.01). The MT group showed a numerically higher baseline NIHSS score [4 (IQR, 2–5) vs. 3 (IQR, 1–4); *P* = 0.06]. Baseline covariates were balanced between the groups following IPTW adjustment.

**Table 1:** Baseline Characteristics of the Study Population.

**Baseline characteristic**	**Unmatched**			**IPTW**		
	**MM (*****n*** = **92)**	**MT (*****n*** = **48)**	***P*** **-value**	**MM(*****n*** = **137.97)**	**MT (*****n*** = **138.40)**	***P*** **-value**
Male, *n* (%)	62 (67.4)	35 (72.9)	0.50	97.4 (70.6)	102.6 (74.1)	0.69
Age, y, mean (SD)	61.9 (10.4)	59.9 (12.2)	0.32	60.3 (11.0)	60.6 (12.1)	0.93
coronary_heart_disease, *n* (%)	20 (21.7)	9 (18.8)	0.68	29.2 (21.2)	30.3 (21.9)	0.94
atrial_fibrillation, *n* (%)	6 (6.5)	6 (12.5)	0.24	10.0 (7.2)	10.1 (7.3)	0.99
hypertension, *n* (%)	62 (67.4)	27 (56.3)	0.19	86.7 (62.9)	89.7 (64.8)	0.85
hyperlipidemia, *n* (%)	8 (8.7)	4 (8.3)	0.94	10.4 (7.5)	7.8 (5.6)	0.65
diabetes, *n* (%)	25 (27.2)	9 (18.8)	0.27	40.3 (29.2)	37.7 (27.2)	0.85
smoking, *n* (%)	28 (30.4)	21 (43.8)	0.12	50.7 (36.7)	52.3 (37.8)	0.92
IVT, *n* (%)	19 (20.7)	7 (14.6)	0.38	26.5 (19.2)	26.7 (19.3)	0.99
Baseline mRS (IQR)	0 (0, 0)	0 (0, 0)	0.33	0 (0, 0)	0 (0, 0)	0.18
Baseline NIHSS (IQR)	3 (1, 4)	4 (2, 5)	0.06	3 (2, 5)	3 (2, 4)	0.84
Baseline ASPECTS (IQR)	8 (8, 9)	8 (7.25, 9)	0.21	8 (7, 9)	8 (8, 9)	0.84
**Occlusion**, ***n*** **(%)**			< 0.01			0.96
ICA	18 (19.6)	24 (50.0)		40.1 (29.1)	42.1 (30.4)	
MCA M1	58 (63.0)	21 (43.8)		79.1 (57.3)	80.4 (58.1)	
MCA M2	16 (17.4)	3 (6.3)		18.7 (13.6)	15.9 (11.5)	
LSW to treatment, minutes	375 (240, 720)	450 (277, 855)	0.27	411.5 (240, 720)	404.7 (180, 720)	0.96
**TOAST subtypes**, ***n*** **(%)**			0.13			0.68
LAA	83 (90.2)	38 (79.2)		123.9 (89.8)	124.2 (89.7)	
CE	8 (8.7)	10 (20.8)		13.1 (9.5)	14.3 (10.3)	
ODE	1 (1.1)	0 (0.0)		1.0 (0.7)	0 (0.0)	

IPTW, inverse probability of treatment weighting; MM, medical management; MT, mechanical thrombectomy; IVT, isolated intravenous thrombolysis; mRS, modified rankin scale; NIHSS, NIH stroke scale; ASPECTS, alberta stroke program early CT score; MCA, middle cerebral artery; ICA, internal carotid artery; LSW, last seen well; LAA, large-artery atherosclerosis; CE, cardioembolism; ODE, other determined.

### Clinical outcomes

**Figure 2:**
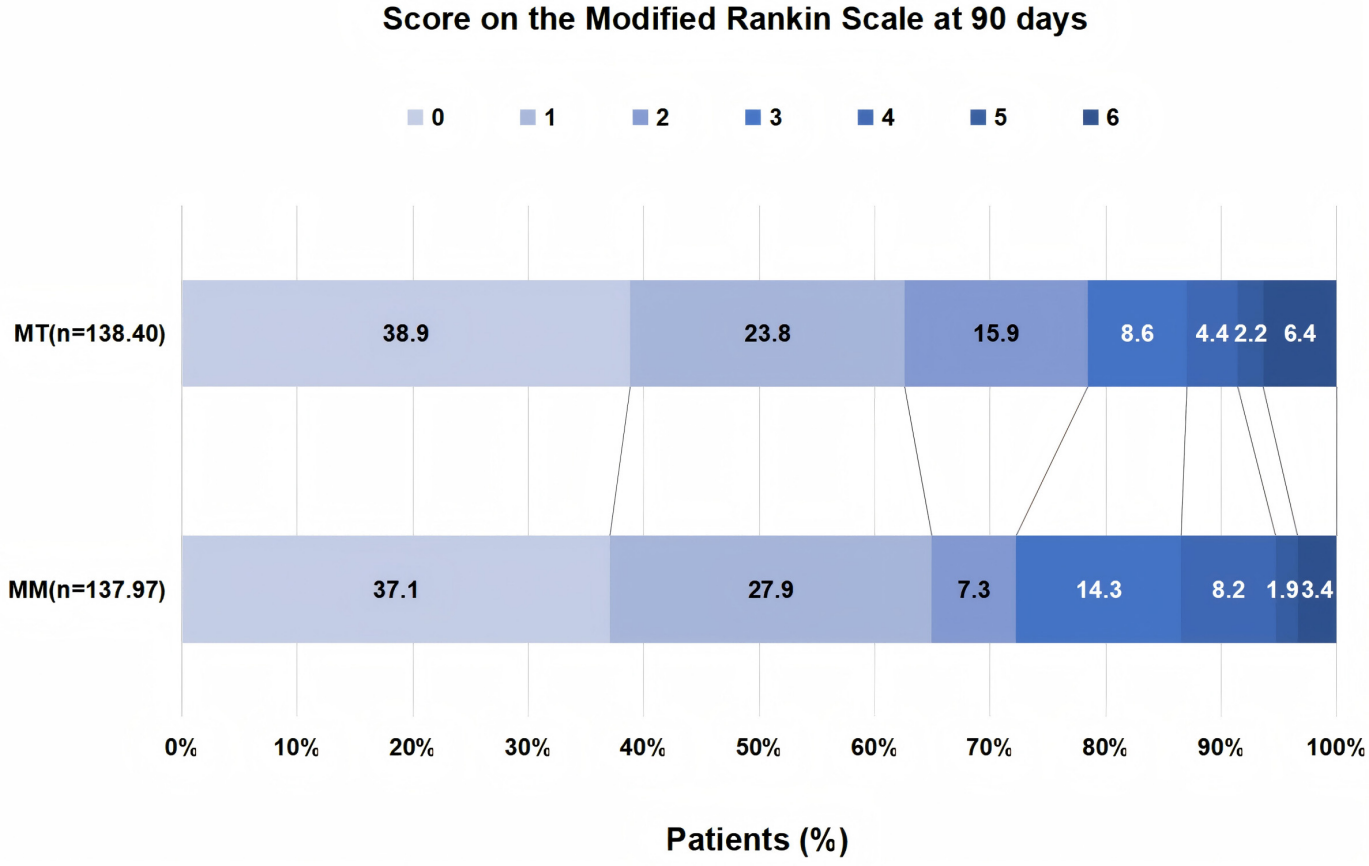
Distribution of 90-day modified rankin scale (mRS) scores based on the IPTW-adjusted cohort. MM, medical management; MT, Mechanical thrombectomy.

The distribution of clinical outcomes between MT and MM is shown in Table 2. The two groups showed comparable results with respect to 3-month excellent outcome (62.5% vs. 65.2%, *P* = 0.75) or functional independence at 3 months (85.4% vs. 71.7%, *P* = 0.07; Table 2). Additionally, the incidence of sICH was numerically higher in the MT group (8.3% vs. 5.4%, *P* = 0.51), but the difference did not reach statistical significance. Mortality rates were low in both groups (2.1% vs. 3.3%, *P* = 0.68). Consistent outcomes were obtained in the IPTW-adjusted model (Table 2, Figure 2).

**Table 2:** Clinical Outcomes and the Association With Mechanical Thrombectomy in Patients With Low NIHSS Scores.

**Outcome**	**Unmatched**			**IPTW**		
	**Event (%)**	**Adjusted OR (95% CI)**	***P*** **-value**	**Event (%)**	**IPTW OR (95% CI)**	***P*** **-value**
**mRS_score_at_90d(0–1)**						
MM	60 (65.2)			89.6 (64.9)		
MT	30 (62.5)	0.93 (0.41–2.11)	0.86	86.7 (62.6)	0.96 (0.41–2.20)	0.92
**mRS_score_at_90d(0–2)**						
MM	66 (71.7)			99.7 (72.3)		
MT	41 (85.4)	2.14 (0.77–5.91)	0.14	108.7 (78.5)	1.55 (0.58–4.12)	0.38
**mortality**						
MM	3 (3.3)			4.6 (3.4)		
MT	1 (2.1)	0.56 (0.02–20.94)	0.75	8.8 (6.4)	1.39 (0.20–9.81)	0.74
**sICH**						
MM	5 (5.4)			8.3 (6.0)		
MT	4 (8.3)	1.63 (0.37–7.14)	0.52	10.6 (7.6)	1.27 (0.30–5.38)	0.75

mRS, modified rankin scale; OR, odds ratio, with the MM group as the reference; CI, confidence interval; IPTW, inverse probability of treatment weighting; MM, medical management; MT, mechanical thrombectomy; MM, medical management; MT, Mechanical thrombectomy; NIHSS, NIH stroke scale; sICH, symptomatic intracranial hemorrhage.

Adjusting sex, age, baseline NIHSS, and Occlusion Site.

In the multivariable analysis adjusted for covariates (Table 2), the two groups showed comparable rates of excellent outcome (aOR = 0.93; 95% CI, 0.41–2.11; *P* = 0.86) and functional independence (aOR = 2.14; 95% CI, 0.77–5.91; *P* = 0.14). Consistent results were observed in the IPTW analysis, which also showed no significant differences in excellent outcomes (IPTW-adjusted OR = 0.96; 95% CI, 0.41–2.20; *P* = 0.92) or functional independence (IPTW-adjusted OR = 1.55; 95% CI, 0.58–4.12; *P* = 0.38).

The rates of sICH (aOR = 1.63; 95% CI, 0.37–7.14; *P* = 0.52; IPTW-adjusted OR = 1.27; 95% CI, 0.30–5.38; *P* = 0.75) and mortality (aOR = 0.56; 95% CI, 0.02–20.94; *P* = 0.75; IPTW-adjusted OR = 1.39; 95% CI, 0.20–9.81; *P* = 0.74) were not significantly different between the two groups in both the covariate-adjusted and IPTW-adjusted analyses.

### Subgroup analyses

Further analysis revealed that, after stratification by time window, no significant association was observed between MT and excellent outcome in either the early window (aOR = 0.52; 95% CI, 0.14–1.94; *P* = 0.33) or the late window (aOR = 1.62; 95% CI, 0.45–5.87; *P* = 0.46; Table 3).

**Table 3:** Excellent outcome rates (mRS 0–1) and unadjusted vs. adjusted odds ratios between MT and MM, stratified by treatment time window.

**LSW to treatment, hours**	**Excellent outcome (mRS, 0–1), %**		**Excellent outcome (mRS, 0–1)**			
	**MT**	**MM**	**OR (95% CI)**	***P*** **-value**	**aOR (95%CI)**	***P*** **-value**
≤ 6	57.9	69.6	0.60 (0.20–1.82)	0.37	0.52 (0.14–1.94)	0.33
>6	65.5	60.9	1.22 (0.46–3.22)	0.69	1.62 (0.45–5.87)	0.46

mRS, modified rankin scale; MM, medical management; MT, mechanical thrombectomy; OR, odds ratio, with the MM group as the reference; CI, confidence interval; LSW, last seen well.

Adjusting sex, age, baseline NIHSS, and Occlusion Site.

## Discussion

Among patients with anterior circulation ELVO and low NIHSS scores, our analysis showed comparable rates of excellent outcomes (62.5% vs. 65.2%) and functional independence (85.4% vs. 71.7%) between the MT and MM groups. Moreover, MT was not associated with higher rates of sICH or mortality at 90 days. The IPTW-adjusted analysis confirmed this finding.

A multicenter study was conducted to compare MT (*n* = 202) and MM (*n* = 116) in the treatment of patients with mELVO (16). They found that, compared with MM, MT was associated with similar rates of excellent outcomes at 90 days (aOR = 0.86; 95% CI, 0.49–1.50; *P* = 0.59) and functional independence (aOR = 0.72; 95% CI, 0.35–1.50; *P* = 0.39). Likewise, there were no statistically significant differences in the risks of intracranial hemorrhage or mortality. These results are consistent with our findings. However, in contrast to their study, which included patients with a prestroke mRS score of ≤ 4, our study restricted enrollment to mELVO patients with a prestroke mRS score of < 2. Our findings are consistent with those reported by Sarraj et al. (17), who observed favorable outcomes in 55.7% and independent outcomes in 63.3% of patients treated with MT. Compared with MM, MT did not demonstrate a significant clinical advantage. Similarly, a multicenter observational study and meta-analysis involving 251 patients reported that mechanical thrombectomy was associated with functional outcomes comparable to those of medical management (MM) in patients with mild stroke (NIHSS < 6) (18). Moreover, the meta-analysis suggested that the difference in symptomatic intracranial hemorrhage disappeared after adjusting for confounding factors (adjusted OR = 2.89; *P* = 0.12).

Additionally, no significant association was observed between MT and favorable 90-day functional outcomes among patients in the late treatment window relative to the early window (aOR = 1.62; 95% CI, 0.45–5.87; *P* = 0.46). Evidence suggests that nearly 60% of patients with ELVO and mild stroke face a high risk of neurological deterioration within 6 h of onset (19). Although our data may suggest that the efficacy of mechanical thrombectomy (MT) in the late time window may not differ significantly from the early time window (Table 3), this finding must be interpreted with caution. The exclusion of patients with neurological deterioration within 6 h in the early time window could lead to an underestimation of the treatment effect. Additionally, in the late time window, the lack of intravenous thrombolysis may result in poorer outcomes, further highlighting the importance of future studies to assess the true impact of delayed treatment on MT efficacy.

Our study included more cases of proximal intracranial occlusions (ICA and MCA-M1). Theoretically, neurological deterioration may be more common in patients with proximal anterior circulation occlusions (20). MT offers prompt and reliable restoration of vessel patency. The potential benefit of MT may have been attenuated by well-compensated collateral circulation in mELVO patients; however, this remains a hypothesis, as collateral status was not evaluated in the present study.

Even though MT was not significantly associated with improved outcomes at 3 months, the possibility of meaningful benefit in specific patient subgroups cannot be excluded. For instance, in patients with imaging evidence of a substantial volume of salvageable ischemic tissue, MT may improve clinical outcomes under specific conditions (21, 22). Future studies should perform stratified analyses to better determine the therapeutic efficacy of MT across relevant patient subgroups.

This study has several limitations. First, this was a retrospective study. Despite using methods like propensity score adjustment to minimize confounding, the influence of unmeasured or unknown confounders cannot be entirely excluded. Thus, the results should be interpreted cautiously. Second, a key limitation of this study is its relatively small sample size (*n* = 140), which may have limited the statistical power to detect significant associations between treatment modality and clinical outcomes. For instance, due to the small number of patients with M2 occlusions, we were unable to conduct detailed stratified analyses by occlusion location, which may have limited the depth of our interpretation. Second, as complete imaging data were not available for all patients, this study was unable to systematically assess collateral status, which limited further exploration of its role in disease progression and prognosis. Future studies incorporating collateral status evaluation could provide additional insights into the impact of collateral circulation on treatment outcomes. Third, NIHSS may not adequately reflect the stroke severity in patients with mild symptoms. Certain deficits such as aphasia or hemianopia can be highly disabling, whereas others like mild facial palsy or slight dysarthria may have minimal functional impact. Treatment decisions and prognostic evaluations could be influenced by this heterogeneity. Fourth, the mRS score, while commonly used to assess functional outcomes, may fail to capture subtle improvements in fine motor or language function among mELVO patients. Future studies may consider using the Barthel Index for a more granular evaluation of 3-month functional independence. Further randomized controlled trials are warranted to establish the safety and efficacy of MT compared to MM in patients with mELVO.

## Conclusions

In conclusion, our multicenter study showed no significant functional or safety benefit of MT in patients with LVO and low NIHSS scores. Future randomized clinical trials are warranted to assess the clinical benefit of mechanical thrombectomy (MT) compared with medical management (MM) in patients with mELVO.

## Data Availability

The datasets presented in this article are not readily available because the data that support the findings of this study are not publicly available but are available from the corresponding author or data sharing committee upon reasonable request. Requests to access the datasets should be directed to Gaopan Zhang, gaopanzhang123@sina.com.
